# Tracing TMEM106B fibril deposition in aging and Parkinson’s disease with dementia brains

**DOI:** 10.1093/lifemedi/lnae011

**Published:** 2024-03-07

**Authors:** Wanbing Zhao, Yun Fan, Qinyue Zhao, Zhen Fan, Jue Zhao, Wenbo Yu, Wensheng Li, Dan Li, Cong Liu, Jian Wang

**Affiliations:** Department of Neurology and National Research Center for Aging and Medicine & National Center for Neurological Disorders, State Key Laboratory of Medical Neurobiology, Huashan Hospital, Fudan University, Shanghai 200040, China; Department of Neurology and National Research Center for Aging and Medicine & National Center for Neurological Disorders, State Key Laboratory of Medical Neurobiology, Huashan Hospital, Fudan University, Shanghai 200040, China; Bio-X Institutes, Key Laboratory for the Genetics of Developmental and Neuropsychiatric Disorders (Ministry of Education), Shanghai Jiao Tong University, Shanghai 200030, China; Department of Neurosurgery, Huashan Hospital, Shanghai Medical College, Fudan University, Shanghai 200040, China; Department of Neurology and National Research Center for Aging and Medicine & National Center for Neurological Disorders, State Key Laboratory of Medical Neurobiology, Huashan Hospital, Fudan University, Shanghai 200040, China; Department of Neurology and National Research Center for Aging and Medicine & National Center for Neurological Disorders, State Key Laboratory of Medical Neurobiology, Huashan Hospital, Fudan University, Shanghai 200040, China; Department of Anatomy and Histoembryology, School of Basic Medical Sciences, State Key Laboratory of Medical Neurobiology and MOE Frontiers Center for Brain Science, Institutes of Brain Science, Fudan University, Shanghai 200032, China; Bio-X Institutes, Key Laboratory for the Genetics of Developmental and Neuropsychiatric Disorders (Ministry of Education), Shanghai Jiao Tong University, Shanghai 200030, China; Zhangjiang Institute for Advanced Study, Shanghai Jiao Tong University, Shanghai 200240, China; WLA Laboratories, World Laureates Association, Shanghai 201203, China; Interdisciplinary Research Center on Biology and Chemistry, Shanghai Institute of Organic Chemistry, Chinese Academy of Sciences, Shanghai 201210, China; State Key Laboratory of Chemical Biology, Shanghai Institute of Organic Chemistry, Chinese Academy of Sciences, Shanghai 200032, China; Department of Neurology and National Research Center for Aging and Medicine & National Center for Neurological Disorders, State Key Laboratory of Medical Neurobiology, Huashan Hospital, Fudan University, Shanghai 200040, China

**Keywords:** TMEM106B fibril, aging, neurodegenerative disease, amyloid aggregation, Parkinson’s disease with dementia

## Abstract

Transmembrane protein 106B (TMEM106B), previously identified as a risk factor in frontotemporal lobar degeneration, has recently been detected to form fibrillar aggregates in the brains of patients with various neurodegenerative diseases (NDs) and normal elders. While the specifics of when and where TMEM106B fibrils accumulate in human brains, as well as their connection to aging and disease progression, remain poorly understood. Here, we identified an antibody (NBP1-91311) that directly binds to TMEM106B fibrils extracted from the brain *in vitro* and to Thioflavin S-positive TMEM106B fibrillar aggregates in brain sections. We discovered that TMEM106B fibrils deposit in the human brain in an age-dependent manner. Notably, the TMEM106B fibril load in the brains of Parkinson’s disease with dementia patients was significantly higher than in age-matched elders. Additionally, we found that TMEM106B fibrils predominantly accumulate in astrocytes and neurons and do not co-localize with the pathological deposition formed by other amyloid proteins such as α-synuclein, Aβ, and Tau. Our work provides a comprehensive analysis of the burden and cellular distribution of TMEM106B fibrils in human brains, underscoring the impact of both aging and disease conditions on TMEM106B fibril deposition. This highlights the potential significance of TMEM106B fibrils in various age-related NDs.

## Introduction

Transmembrane protein 106B (TMEM106B), a type II endolysosomal membrane protein, is associated with endolysosome morphology, location, acidification, and trafficking [1–3]. It was identified as a contributing factor to frontotemporal lobar degeneration (FTLD), particularly in individuals with progranulin (*GRN*) mutations [4, 5]. *TMEM106B* genetic variations are known to influence a broad spectrum of clinical and pathological characteristics in FTLD, such as the timing of disease onset and mortality, cognitive abilities, and the presence of neuroastroglial tauopathy [6–9]. Additionally, TMEM106B is implicated in the pathology of various other neurodegenerative diseases (NDs), extending beyond FTLD. For Alzheimer’s disease (AD), it acts as both a disease risk modifier and an influencer of clinical and pathological characteristics [10–13]. While it does not directly trigger disease development, TMEM106B is linked to TAR DNA-binding protein (TDP-43) pathology and cognitive issues in amyotrophic lateral sclerosis (ALS) [14–16]. It also contributes to cognitive decline progression in Parkinson’s disease (PD) and is associated with brain volume, neuron count, and cognition in normal aging [6, 17–19].

Amyloid fibrillar aggregates, common pathological hallmarkers in different NDs, accumulate abnormally in the human diseased brain. These fibrillar aggregates include amyloid β (Aβ) fibrils and Tau-composed neurofibrillary tangles (NFT) in AD [20–22], α-synuclein (α-syn) fibrils in PD [23–25], and TDP-43 fibrils in FTLD and ALS [26–28]. These different amyloid proteins form fibrils in various cell types and brain regions, each contributing to distinct clinical pathologies in NDs [29–32]. Additionally, different amyloid fibrils co-exist and interact in ND-affected brains, potentially accelerating disease progression [33]. Significantly, the luminal domain of TMEM106B (residues120–254) was recently discovered to form extensive amyloid fibrils in brains affected by various NDs and in normal aging [34–37], suggesting its critical involvement in disease and aging. However, the cellular location and distribution of TMEM106B fibrils, and their relationship with other amyloid aggregates in NDs brains, remain unknown.

In this work, we identified NBP1-91311 as a reliable antibody for directly binding to TMEM106B fibrils extracted from brain tissues of patients with Parkinson’s disease with dementia (PDD) and a 101-year-old normal elder. We further confirmed NBP1-91311’s binding to TMEM106B fibrils in brain sections through double-staining with a classical amyloid fibril dye–Thioflavin S (ThS). Notably, we found that fibrillar TMEM106B accumulation is age-dependent and exacerbated by disease conditions. TMEM106B fibrils were found in a variety of cell types without co-localization with fibrillar aggregates formed by other pathogenic amyloid proteins. Our findings systematically characterize the burden and distribution of TMEM106B fibrils and suggest that both aging and NDs may promote the deposition of these fibrils.

## Results

### NBP1-91311 antibody directly binds to brain-extracted TMEM106B fibril

**Figure 1.**
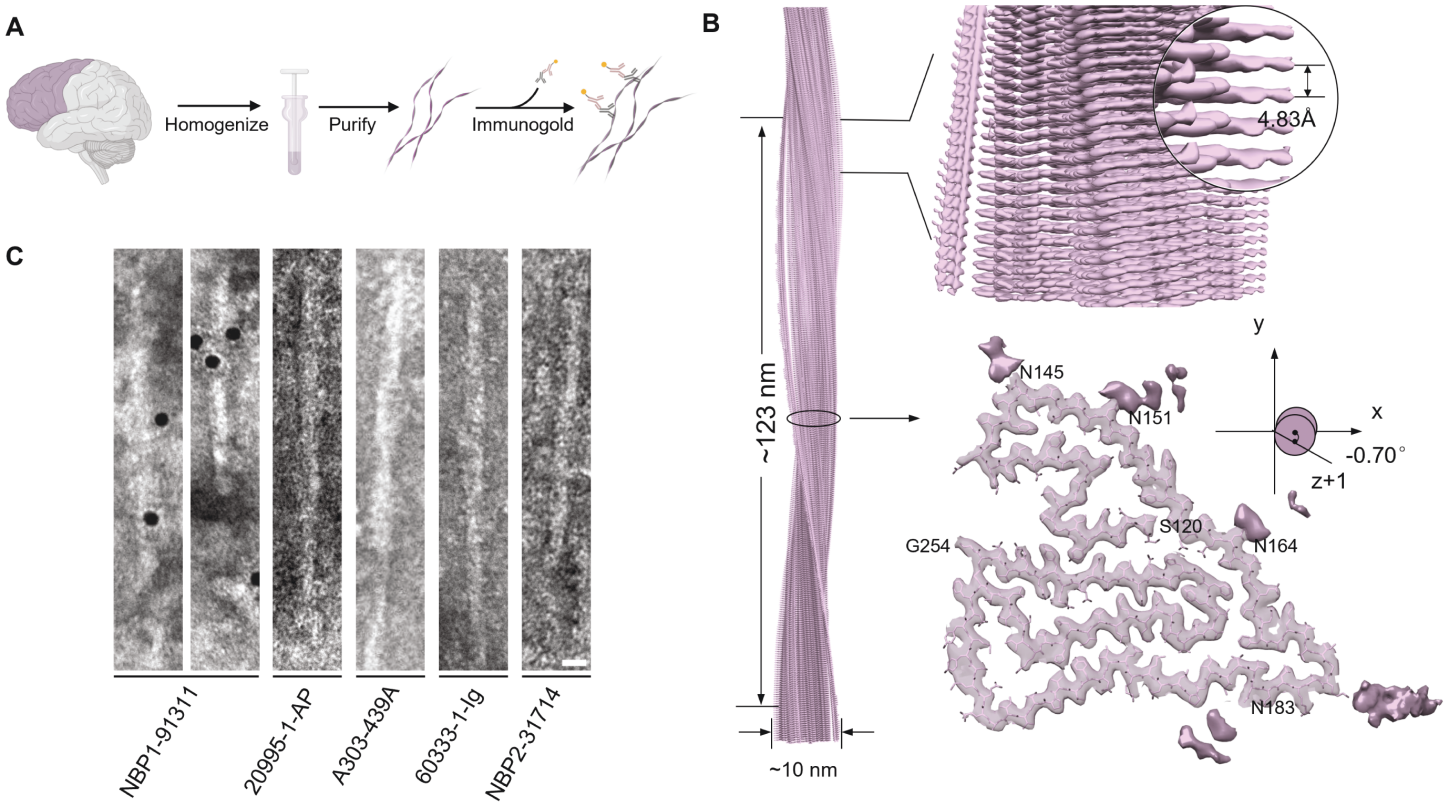
**NBP1-91311 antibody can directly bind to *ex vivo* TMEM106B fibrils.** (A) Workflow of the extraction and immunogold staining of TMEM106B fibrils from the frontal lobe of case 1 of PDD. (B) Cryo-EM density map and structural model (top view) of the Type 1 fibril extracted from case 1 of PDD. Fibril width, length of half pitch, helical rise, and twist angle are indicated. The twist angle is graphically illustrated. Graphing was performed with UCSF Chimera v1.13. The terminal residues and four potentially glycosylated Asn residues are labeled. (C) Representative images of immunogold staining of Type 1 TMEM106B fibril extracted from case 1 of PDD using five commercial antibodies. Scale bar, 50 nm.

To map out the morphological characteristics and distribution of TMEM106B fibrils in the human brain, we aimed to identify a TMEM106B-specific antibody that could directly bind to TMEM106B fibrils. Utilizing our established protocol for fibril extraction [34], we successfully isolated a significant quantity of amyloid fibrils directly from the frontal cortex of the case 1 of PDD (Figs. 1A and S1A, Table 1). Utilizing cryo-electron microscopy (cryo-EM), we determined the structure of brain-extracted fibril at a resolution of 3.5 Å (Figs. 1B, S1B and S1C, Table S1). The cryo-EM analysis revealed a singular, predominant fibril morphology within the fibril sample extracted from the brain, designated as Type 1 fibrils, which corresponds to the Type 1 TMEM106B fibril structure as we previously reported (Fig. S1D) [34]. This particular TMEM106B fibril core is composed of the residues 120–254, which folds into a curling stone-like structure (Fig. 1B).

**Table 1.** Overview of the subjects used in this study

Conditions	Case number	Sex	Age (yo)	Cause of death	Pathology	Brain regions
Non-ND controls	1^a^	F	35	NA	NA	Temporal lobe
	2	F	43	Abdominal tumor	NA	Temporal lobe
	3^a^	M	52	NA	NA	Temporal lobe
	4	M	71	Not reported	NA	Parietal lobe
	5	F	97	Ventricular hemorrhage	Aβ aggregates	Temporal lobe
	6	M	101	Old age	Aβ and limited Tau aggregates	Temporal lobe
Parkinson’s disease with dementia	1	M	83	Pneumonia	α-Syn and limited Tau aggregates	Frontal lobe
	2	F	70	Colon cancer with systematic metastasis	α-Syn aggregates	Temporal lobe

Aβ, amyloid β; F, female; M, male; NA, not applicable; non-ND, non-neurodegenerative diseases; yo, years old.

^a^The brain samples of the two controls who undergoing surgery because of brain trauma and parenchymal hemorrhage, respectively, were the abandoned brain tissue produced in the surgery.

We selected five commercially available TMEM106B antibodies, including two (20995-1-AP, NBP1-91311) with immunogens within the fibril-forming C-terminal domain (CTD) of TMEM106B, and three (A303-439A, 60333-1-Ig, NBP2-31714) targeting the N-terminal fragment (NTF) of TMEM106B (Table 2). Through immunogold-staining, we examined whether any of these antibodies could directly bind to the brain-extracted TMEM106B fibrils. Remarkably, only the NBP1-91311 antibody, but not the other four, was able to bind to TMEM106B fibrils (Fig. 1C). To corroborate this finding, we also extracted TMEM106B fibrils from two other brain tissues, one from case 2 of PDD patient and the other from case 6 of non-neurodegenerative disease (non-ND) control (Table 1), corresponding to PDD case and normal 2 case in our previously study [34], respectively. Consistently, only the NBP1-91311 antibody, but not the others, bound to both Type 1 TMEM106B fibril from case 6 of non-ND subject and Type 2 TMEM106B fibril from case 2 of PDD (Fig. S2). Our results demonstrate that the NBP1-91311 antibody can directly bind to TMEM106B fibrils and might be suitable for detecting TMEM106B fibrillar aggregates in human brains.

**Table 2.** Commercial antibodies tested in this study

Source	Cat. No.	Host	Immunogen	Clonality
Novus Biologicals	NBP1-91311	Rabbit	204–255 aa	Polyclonal
Proteintech	20995-1-AP	Rabbit	150–274 aa	Polyclonal
Bethyl Laboratories	A303-439A	Rabbit	1–50 aa	Polyclonal
Proteintech	60333-1-Ig	Mouse	1–50 aa	Monoclonal (5D1F8)
Novus Biologicals	NBP2-31714	Rabbit	2–53 aa	Polyclonal

### Characterization of fibrillar TMEM106B in brain tissues

**Figure 2.**
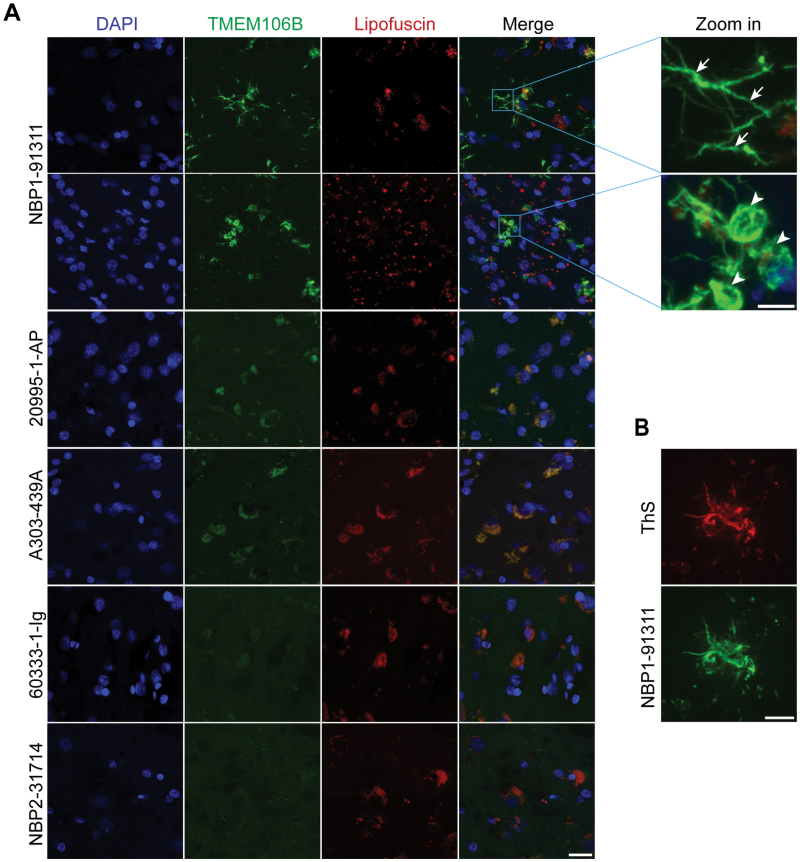
**Visualizing TMEM106B fibrils in brain sections.** (A) Representative images of IF staining of the brain sections of the frontal lobe from case 1 of PDD patient using five commercial antibodies designed for targeting TMEM106B. TMEM106B fibrillar aggregates in the morphology of filamentous processes indicated by white arrows and circle-like condense inclusions indicated by white arrow heads were zoomed in. The intensity of autofluorescent lipofuscin was captured in a separate channel (red). (B) Sequential staining of ThS and TMEM106B using NBP1-91311 of the same brain region as (A). Scale bar, 20 μm (A); 5 μm (Zoomed in view in (A) and (B)).

We proceeded to investigate the potential of the NBP1-91311 antibody for visualizing TMEM106B fibrils in brain tissue sections, using the frontal cortex from case 1 of PDD. By using immunohistochemistry (IHC) staining, we firstly confirmed a varied collection of Lewy bodies and Lewy neurites in this PDD brain but did not observe any Aβ plaques or phosphorylated Tau (p-Tau) pathologies (Fig. S3). To enhance epitope exposure for the immunofluorescence (IF) staining of TMEM106B fibrils, we pretreated the brain sections with formic acid (FA) before applying the primary antibodies. This treatment allowed us to observe numerous locally condensed TMEM106B fibrils signals using the NBP1-91311 antibody with potential interference from autofluorescent lipofuscin that progressively increases over the lifespan and is the most abundant pigment in human brain eliminated [38]. In stark contrast, the other four TMEM106B antibodies, which did not bind to TMEM106B fibrils in our *in vitro* assays, showed no such condensed TMEM106B-positive signals in the brain sections (Fig. 2A). To ensure that these results were not due to unsuitable staining methods, we modified our IF staining protocol by incorporating heat pretreatment with sodium citrate for antigen retrieval, yet the outcomes remained consistent with those from FA pretreatment (Fig. S4).

Furthermore, we observed two distinct morphologies of TMEM106B-positive signals in IF staining using the NBP1-91311 antibody: one resembled as filamentous processes, and the other appeared circular inclusion with a lightly colored core (Fig. 2A). To validate that these intensities stained by NBP1-91311 were indeed TMEM106B fibrillar aggregates, we performed double staining with ThS–a widely used fluorescent amyloid fibril dye [39, 40], alongside NBP1-91311 on the same brain slice. The co-localization of the NBP1-91311 positive signals with the ThS positive signals affirmed that the intensely stained areas by NBP1-91311 represent TMEM106B fibrillar aggregates (Fig. 2B).

### TMEM106B forms fibrils in an age- and disease-dependent manner

**Figure 3.**
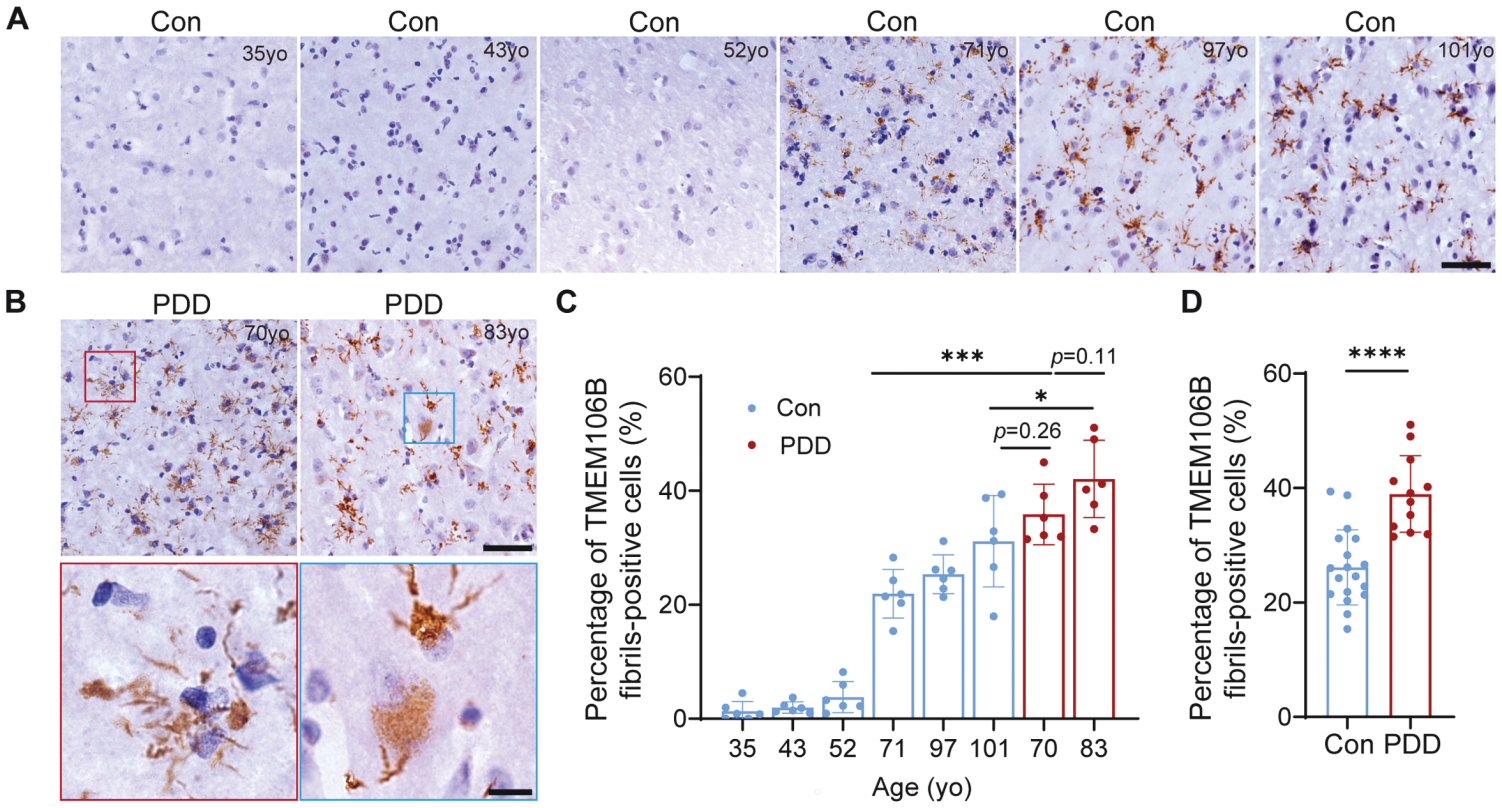
**Characterization of fibrillar TMEM106B deposited in different human brains of eight individuals.** IHC staining of TMEM106B fibrillar aggregates using NBP1-91311 antibody of the brain sections from six non-ND controls with different ages (A) and two PDD patients (B). TMEM106B fibrillar aggregates in the morphology of filamentous processes and cytoplasmic inclusions in neuron and glia in (B) were zoomed in. (C) Statistical analysis of percentage of TMEM106B fibrils-positive cells in six non-ND controls and two PDD patients. Data are expressed as mean ± SD of six images randomly captured from brain slices of each subject. (D) Comparison of the overall burden of TMEM106B fibrillar aggregates in the brain sections of three non-ND controls aged over 70 and two PDD patients. Data in Con group are expressed as mean ± SD of 18 images randomly captured from brain slices of case 4 (71 years old), 5 (92 years old), and 6 (101 years old) of non-ND controls. Data in PDD group are expressed as mean ± SD of 12 images randomly captured from brain slices of case 1 (83 years old), and 2 (70 years old) of PDD patients. Statistical significance in (C, D) was measured using unpaired *t* test. *, *P* < 0.05, ***, *P* < 0.001, ****, *P* < 0.0001. Scale bar, 50 μm (A and B, top); 10 μm (B, bottom).

We next conducted a detailed investigation of TMEM106B fibril prevalence in several human brain samples across various age groups. The IHC analysis included brain sections from six non-ND individuals, whose ages ranged from 35 to 101 years (Table 1). The data revealed that the presence of TMEM106B fibrillar aggregates, detected by NBP1-91311 antibody, significantly escalates with age, indicating a clear age-related pattern (Fig. 3A). For example, the youngest non-ND control subject, aged 35, showed no TMEM106B fibrils. Similarly, very sparse TMEM106B fibril presence was found in the non-ND controls aged 43 and 52. In contrast, a pronounced and widespread distribution of TMEM106B fibrils was present in subjects aged 71 to 101.

We proceeded to evaluate the TMEM106B fibril load in the brains of two PDD patients of differing ages, comparing these results with age-matched non-ND individuals. The TMEM106B fibril accumulation appears to be slightly higher in the 83-year-old PDD patient compared to the 70-year-old PDD patient, though without statistical significance (*P *= 0.11) (Fig. 3B and 3C). Strikingly, the burden of TMEM106B fibrillar aggregates in the 70-year-old PDD patient was significantly more pronounced than in the 71-year-old non-ND individual. Moreover, the TMEM106B fibril levels in the 83-year-old PDD patient were found to be remarkably severe in comparison with those in the 101-year-old elder (Fig. 3B and 3C). Collectively, the overall burden of TMEM106B fibrils in the two PDD patients was far more severe than that in the three non-ND controls aged over 70 (Fig. 3D). These findings underscore that the accumulation of TMEM106B fibrils in the human brain is not only age-dependent but also markedly exacerbated by the pathology of PDD.

### Cellular distribution of fibrillar TMEM106B and its association with other pathological amyloid fibrils

**Figure 4.**
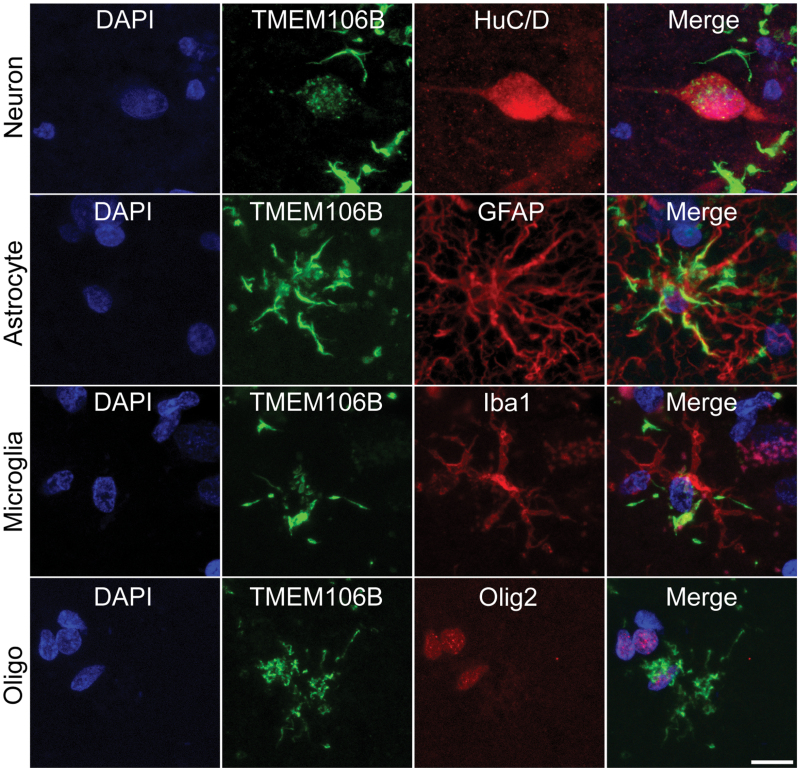
**Cellular localization of fibrillar TMEM106B.**Double IF staining of fibrillar TMEM106B and HuC/D (neuron marker), GFAP (astrocyte marker), Iba1 (microglia marker), and Olig2 (oligodendrocyte marker), respectively. The brain sections of the temporal lobe from case 6 of non-ND individual were used. Scale bar, 10 μm.

Similar to the observation of IF staining (Fig. 2A), IHC staining also indicated that TMEM106B fibrillar aggregates, positive for the NBP1-91311 antibody, mainly exhibited two morphologies: dense cytoplasmic inclusions and filamentous processes within neuronal and glial cells (Fig. 3B, bottom). Thus, we finally sought to delineate the cellular distribution and morphological characteristics of TMEM106B fibrillar aggregates in brain tissues and to understand their association with other pathological fibrillar aggregates commonly found in NDs. Using double IF staining, we examined the temporal cortex from case 6 of non-ND individual for TMEM106B fibrils and classic cellular markers of neurons (Hu C/D) and glia (GFAP for astrocytes, Iba1 for microglia, and Olig2 for oligodendrocytes) (Fig. 4). The results showed that neurons positive for HuC/D showed dense, coarse cytoplasmic TMEM106B inclusions, while astrocytes marked by GFAP primarily contained filamentous TMEM106B structures. In Iba1-positive microglia, both cytoplasmic inclusions and filamentous processes of TMEM106B were present, albeit in smaller quantities compared to astrocytes. Additionally, TMEM106B-positive inclusions were detected surrounding the nuclei of oligodendrocytes, as identified by Olig2 staining, particularly in the subcortical white matter. These findings imply that TMEM106B forms distinct morphological fibrillar aggregates, predominantly depositing in neurons and astrocytes, and to a lesser extent in microglia and oligodendrocytes.

**Figure 5.**
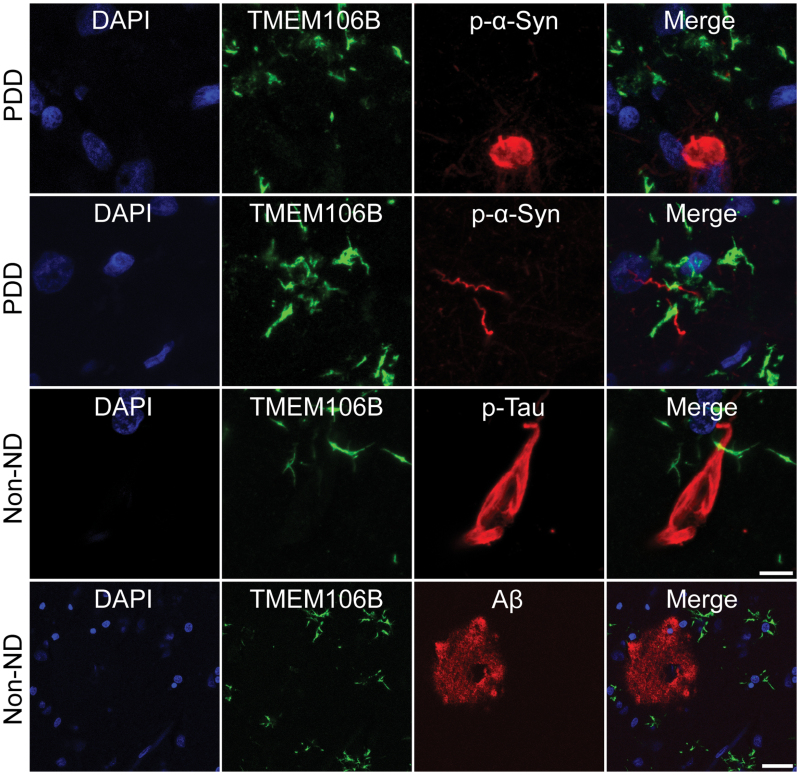
**No co-localization between fibrillar TMEM106B and fibrillar aggregates formed by α-syn, Tau, and Aβ.** Double IF staining of TMEM106B fibrils and fibrillar aggregates formed by p-α-Syn, p-Tau, and Aβ, respectively. No co-localization of fibrillar TMEM106B and Lewy bodies and Lewy neurites in brain sections from case 2 of PDD patient, as well as neurofibrillary tangles and Aβ plaques in brain sections from case 6 of non-ND individual. Scale bar, 30 μm in TMEM106B and Aβ double staining, 8 μm in others.

Further, to explore the association between TMEM106B fibrils and other pathological fibrillar aggregates, we conducted double IF staining of TMEM106B fibril with other fibrillar aggregates identified in NDs, including phosphorylated α-syn (p-α-syn), p-Tau, and Aβ (Fig. 5). When examining the brain tissue of case 2 of PDD, we found that TMEM106B did not co-localize with α-syn fibrillar aggregates, including Lewy bodies and Lewy neurites. Similarly, in the brain slice from case 6 of non-ND subject, there was no observed co-localization of TMEM106B with either p-Tau or Aβ plaques. Therefore, it appears that TMEM106B fibrils do not co-aggregate with these pathological amyloid proteins in brains affected by NDs.

## Discussion

TMEM106B was firstly identified as a risk factor for developing frontotemporal lobar degeneration with TDP-43 inclusions (FTLD-TDP) through a genome-wide association study in 2010 [4]. Since then, the past decade has seen a culmination of research into the role of TMEM106B in FTLD and other NDs [41]. Initially, the focus was largely on genetic studies linking TMEM106B haplotypes with disease risk and various clinical and pathological phenotypes of NDs [6, 10, 14, 16]. Significantly, TMEM106B genetic variations have been found to not only influence the risk of developing FTLD, AD [4, 5, 10, 11], hippocampal sclerosis of aging [42, 43], and limbic-predominant age-related TDP-43 encephalopathy (LATE) [44, 45], but also associated with the progression of multiple NDs [6, 16]. Mouse models deficient in *GRN*, when also knocked out *TMEM106B*, show exacerbated lysosomal dysfunction and FTLD pathologies [46–48]. Conversely, some studies suggest that loss-of-function of TMEM106B can mitigate these deficits [49]. Thus, the precise physiological and pathological roles of TMEM106B remain elusive.

Recently, several groups have successfully isolated a significant number of TMEM106B fibrils from the brains of patients with diverse NDs and individuals undergoing normal aging processes, underscoring the potential critical role of TMEM106B fibrils in both NDs and aging [34–37]. Despite this progress, there is ongoing debate about the exact role and importance of TMEM106B fibrils in these contexts. For instance, Goedert and colleagues have proposed that the formation of TMEM106B fibrils is predominantly age-dependent and occurs irrespective of disease states [35]. While the Eisenberg group posits that TMEM106B fibrils might be a primary pathogenic factor in FTLD-TDP [36]. Our own prior research suggests that the development of TMEM106B fibrils is influenced by both the aging process and disease conditions [34]. However, the precise timing and anatomical locations for the formation of TMEM106B fibrillar aggregates in the brain remain unclear, as is the interaction between TMEM106B fibrils and other well-characterized pathological fibrillar aggregates during the development and progression of NDs.

Two antibodies, TMEM239 from Goedert’s lab and SB0051 from Rademakers’s lab, were developed against a peptide sequence within CTD of TMEM106B [35, 50], a region involved in the fibril core. Despite their potential for characterizing brain TMEM106B fibrils, it is unknown if they could directly bind to the fibrils, and issues with Western blot and IHC detection of TMEM106B aggregates have been noted [51]. Additionally, these lab-generated antibodies are not widely accessible. In this study, we screened various commercial TMEM106B antibodies for their ability to directly bind to brain-extracted TMEM106B fibrils and identified the NBP1-91311 antibody as effective for detecting TMEM106B fibrils both *in vitro* and in brain samples. Using NBP1-91311, we further characterized TMEM106B fibrils in brain sections from eight subjects with different ages and disease conditions.

We observed that TMEM106B fibrils primarily deposit in neurons and astrocytes and exhibit distinct morphologies in different cell types. Consistent with the finding of TMEM106B inclusions visualized by TMEM239 and TMEM106B C-terminal fragment aggregates stained by SB0051 [35, 50], we noted an age-dependent increase in TMEM106B fibril burden with unique morphological characteristics. Furthermore, we observed that TMEM106B fibril loads in PDD patients were more substantial than those age-matched non-ND individuals. This aligns with recent studies showing greater TMEM106B aggregation in FTLD or LATE patients compared to age-matched healthy elders [50, 52]. However, we did not find co-localization of TMEM106B fibrils with well-known pathological amyloid aggregates formed by p-α-syn, Tau, and Aβ. Further investigation is crucial to decipher the pathological mechanisms behind TMEM106B fibrillization in NDs.

**Figure 6.**
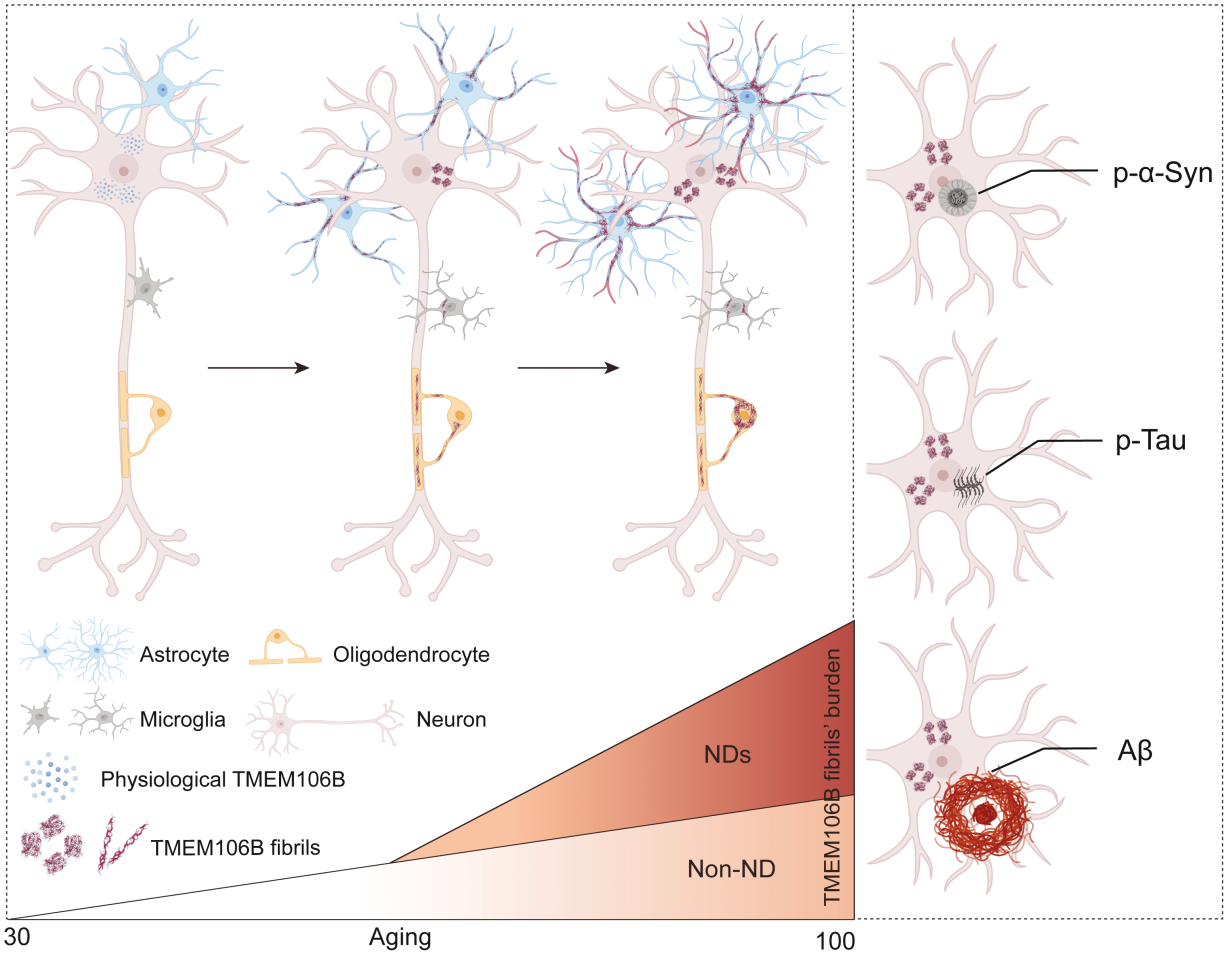
**Overview of characterization of TMEM106B fibrils’ burden, distribution, and relationship with other well-identified amyloid proteins in NDs in human brain.**The overall burden of TMEM106B fibrils that predominantly accumulate in neurons and astrocytes increase along aging and further exacerbate under NDs pathological conditions (left). No co-localization between TMEM106B fibrils and other amyloid fibrillar aggregates, including Lewy bodies and Lewy neurites consisted of p-α-Syn, p-Tau-composed neurofibrillary tangles, and Aβ plaques, was found (right).

In conclusion, our study confirmed that one commercially accessible antibody, NBP1-91311, can directly recognize TMEM106B fibrils both *in vitro* and in brain samples. We further characterized TMEM106B fibrils formed in human brains from eight individuals using this identified antibody. Results showed that TMEM106B fibril formed in an age-dependent and disease-condition exacerbated manner and distributed predominately in neurons and astrocytes but not co-localized with other amyloid proteins (Fig. 6). Our findings suggest that both age and disease conditions are stimulators for TMEM106B fibrils formation in human brain, which implies the potential pivotal roles of TMEM106B fibrilization in the clinicopathogenesis of a multitude of age-associated NDs.

## Research limitations

Overall, our study comprehensively and directly characterizes the burden and distribution of TMEM106B fibrils within human brain tissue, using an antibody (NBP1-91311) that we have identified for its unique ability to bind to the TMEM106B fibrils, and proposes that both aging and disease conditions may facilitate the abnormal aggregation of these fibrils. However, though we revealed that the burden of TMEM106B fibrils in PDD is far more severe than that in age-matched non-ND controls, whether the same results could be found in other NDs, such as AD, FTLD, remains to be explored. Additionally, future studies should further explore the distribution pattern of TMEM106B fibrillar aggregates in different age and brain regions of normal elders, as well as NDs, as Braak staging of α-syn pathology in PD and Tau pathology in AD did [20, 23]. Given TMEM106B fibrils present in the brains of not only NDs but also normal elders, the pathological role of TMEM106B fibrils is still uncertain. Whether TMEM106B fibrils possess neurotoxicity and lead to neurodegeneration, as other pathological amyloid fibrils do, is urgent to be investigated.

## Methods

### Human brain samples

Brain samples from eight subjects, including six non-ND controls and two PDD patients, were used in this study (Table 1). Case 1 of non-ND control was a 35-year-old female who had a brain surgery due to brain trauma without neuropathological abnormalities. Case 2 of non-ND control was a 43-year-old female who died due to infection after surgery for abdominal tumors. Case 3 of non-ND control was a 52-year-old male who underwent brain surgery because of cerebral hemorrhage. Case 4 of non-ND control was a 71-year-old male with the death reason not recorded. Cases 1 to 4 of non-ND controls were confirmed to be absent of neuropathological abnormalities. Case 5 of non-ND control was a 97-year-old female who died of ventricular hemorrhage. Abundant Aβ plaques were found in her brain sections of temporal lobe. Case 6 of non-ND control died of old age was a 101-year-old male. This case showed plentiful Aβ and limited Tau aggregates in temporal lobe as we previously reported [34]. Case 5 and 6 of non-ND controls were free of any known NDs. Case 1 of PDD was an 83-year-old male with over 30 years of history of PD and died of severe pneumonia. Case 2 of PDD with 20-year history of PD had a definite family history, but no definite pathogenic gene mutation was detected by whole genome sequencing. We described her clinical and pathological characteristics in our previous studies [34, 53]. We described case 4 and 6 of non-ND controls (corresponding to Normal 1, Normal 2, respectively) and case 2 of PDD before in our previous study [34].

### Research ethics

The brain samples of cases 1 and 3 of non-ND controls were acquired from Brain Disease Biobank of Huashan Hospital. These individuals’ family members gave his or her informed consent for the collection of the abandoned brain tissue produced in the surgery. The protocol used was approved by Ethics Committee of Huashan Hospital Affiliated to Fudan University (the project number KY2020-065). The brain samples of the remaining six subjects were acquired from the Body Donation Station in Fudan University (Shanghai Red Cross Society). Informed consent was obtained from the six donors and their next of kin.

Ethical approval of this study was granted by the Human Studies Institutional Review Board, Huashan Hospital, Fudan University. All procedures carried out in this study were in conformity with the ethical standards of the Declaration of Helsinki.

### Brain sections

For the following immunostaining, the brain tissues of all cases were post-fixed in 4% paraformaldehyde and then were dehydrated in a gradient in sucrose/PBS solution (20%, 30%, 30%, pH 7.4). Next, brain tissues were rapidly frozen with OCT-embedded compounds (Sakura) and serially sectioned in a thickness of 25 μm with a cryostat microtome (Leica).

### IHC

IHC was performed as previously described with slight modifications [34, 53]. In brief, brain sections were pretreated for antigen retrieval using 88% FA for the staining of TMEM106B fibrillar aggregates by anti-TMEM106B (NOVUS, Cat. NBP1-91311), p-α-Syn, p-Tau, and Aβ. Then, brain sections were subjected to 3% hydrogen peroxide solution to quench endogenous peroxidases followed by blocking with 5% bovine serum albumin (BSA) plus 0.3% Triton X-100. Sections were incubated with a primary antibody against C-terminal of TMEM106B (NOVUS, Cat. NBP1-91311; 1:200), p-Tau (Invitrogen, Cat. MN1020; 1:500), p-α-Syn (Abcam, Cat. ab51253; 1:500), or Aβ (BioLegend, Cat. 803015; 1:500) for 12–16 h at 4°C. After that, the sections were sequentially incubated with a biotin-conjugated secondary anti-mouse antibody (Vector, Cat. BA-9200; 1:1000) or anti-rabbit antibody (Vector, Cat. BA-1000; 1:1000) for 2 h and avidin-biotin complex (Vector, Cat. PK-6100; 1:1000) for 1.5 h at room temperature (RT). After labeling using a DAB-peroxidase substrate (Vector, Cat. SK-4100), the sections were counterstained with hematoxylin. Images were captured by the DP74 digital camera connected to an Olympus microscope (Olympus).

### IF staining

For antibody screening, five commercial anti-TMEM106B antibodies were evaluated by IF staining (Table 2) using the frontal lobe from case 1 of PDD patient. For IF of TMEM106B fibrillar aggregates by anti-TMEM106B (NOVUS, Cat. NBP1-91311), antigen retrieval was performed with FA. For the other four antibodies, FA and heating retrieval in citrate antigen retrieval solution were used respectively to fully test the staining efficacy of these antibodies. After antigen retrieval, the brain sections were blocked and permeabilized (5% BSA, 0.3% Triton X-100 in PBS) for 30 min. Primary antibodies of TMEM106B (Table 2) were incubated overnight at 4°C, followed by the incubation of secondary fluorescence antibodies Alexa Fluor 488 (ThermoFisher Scientific; 1:500) at RT for 2 h. Finally, brain sections were mounted in a microscope slide using a mounting medium containing DAPI (SouthernBiotech, Cat. 0100-20,) and scanned with SP8 confocal microscope.

For double-labeling IF staining, brain sections from case 6 of non-ND subject were used. Antigen retrieval was carried out using FA and heating as mentioned above. Then, brain sections were incubated with primary antibodies anti-TMEM106B (NOVUS, Cat. NBP1-91311; 1:100) plus HuC/D (Invitrogen, Cat. A-21271; 1:250), GFAP (Sigma-Aldrich, Cat. G3893; 1:250), Iba1 (Abcam, Cat. ab5076; 1:500), Olig2 (Sigma-Aldrich, Cat. MABN50; 1:250), Aβ (Biolegend, Cat. SIG-39320; 1:500), p-Tau (Invitrogen, Cat. MN1020; 1:300), or p-α-syn (Abcam, ab184674; 1:500), respectively. After that, brain sections were incubated with secondary fluorescence antibodies Alexa Fluor 488,568 or 633 (ThermoFisher Scientific; 1:500) at RT, followed by the treatment with Sudan black to reduce autofluorescence of lipofuscin [54]. Finally, mounting and image captures were conducted as described above.

### ThS and TMEM106B sequential staining

For labeling of ThS, brain sections of case 1 of PDD were incubated in 0.05% ThS (dissolved in 50% ethanol) at RT for 8 min. The sections were rinsed with 50% ethanol for 5 min, dipped into distilled water twice for 3 min, and mounted in ProLong Glass Antifade Mountant (Invitrogen, Cat. P36984). Fluorescence images for ThS were captured using SP8 confocal microscope. After that, all sections labeled with ThS were washed in PBS to remove the mounting media and pretreated with FA. Then TMEM106B antibody (NOVUS, Cat. NBP1-91311; 1:100) and secondary antibody Alexa Fluor 568 (ThermoFisher Scientific; 1:500) was used for TMEM106B labeling. Images were captured using SP8 confocal microscope.

### Extraction of TMEM106B fibrils

The extraction of TMEM106B fibrils from postmortem brain tissues was performed as we previously reported [34]. Briefly, frozen brain tissues, including temporal lobe from case 6 of non-ND control and case 2 of PDD patient, frontal lobe from case 1 of PDD patient, were manually homogenized for three times in extraction buffer (10 mM Tris–HCl pH 7.5, 0.8 M NaCl, 10% sucrose, 1 mM EGTA, 0.1% sarkosyl, PMSF and cocktail). Homogenates were brought to 2% sarkosyl and incubated at 37°C for 1 h with manually shaking after every 15 min for several times, followed by centrifugation at 10,000 *g* for 10 min at 4°C. Then, the supernatants (S1) were subjected to ultracentrifugation at 100,000 *g* for 60 min at 4°C. The pellets (P2) after the ultracentrifugation were collected and resuspended with extraction buffer (1,000 μL/g) following with a low-speed centrifugation at 3,000 g for 5 min at 4°C. The supernatants (S3) were diluted into 3-fold with buffer consisting of 50 mM Tris–HCl (pH 7.5), 0.15 M NaCl, 10% sucrose, and 0.2% sarkosyl and centrifugated at 166,000 g for 1 h at 4°C. The sarkosyl-insoluble pellets (P4) containing TMEM106B fibrils were resuspended with 20 mM Tris-HCl (pH 7.4) and 50 mM NaCl (100 μL/g).

### Negative staining transmission electron microscopy (NS-TEM)

5 μL of resuspended sarkosyl-insoluble pellet was first incubated with pronase for 30 min and then loaded onto the glow-discharged 230 mesh carbon-coated copper grids (Beijing Zhongjingkeyi Technology Co., Ltd.) for 45 s. After blotting the sample with filter paper, 5 μL ddH_2_O and 5 μL 2% (*w*/*v*) uranyl acetate were used sequentially to wash the grid. An additional 5 μL 2% (*w*/*v*) uranyl acetate was applied to stain the sample for 45 s. Sample imaging was performed with a Tecnai T12 microscope (FEI Company) operating at 120 kV.

### Immuno-gold negative-staining electron microscopy

Immuno-gold negative staining electron microscopy was carried out as we described [34]. Briefly, 5 µL of the final pellet containing *ex vivo* TMEM106B fibrils was incubated with pronase for 30 min and loaded onto the glow-discharged 230 mesh carbon-coated copper grids (Beijing Zhongjingkeyi Technology Co., Ltd.) for 2 min. The sample was washed twice with PBS and placed in a blocking buffer (0.1%BSA in PBS) for 10 min at RT, following incubation with TMEM106B antibody (NOVUS, Cat. NBP1-91311; 1:25) for 2 h at RT. Then the grid was washed with PBS twice and was incubated with secondary antibody labeled with 6 nm colloidal gold (Jackson Immuno Research, Cat. 115-195-146; 1:50) for 1 h. After sequentially washing with ddH_2_O twice and 2% (*w*/*v*) uranyl acetate, the grids were stained with 2% (*w*/*v*) uranyl acetate for 45 s. The excess buffer was removed with filter paper. The sample was dried with an infrared lamp, and images were acquired as described above.

### Cryo-EM sample preparation and data collection

After pretreatment with pronase for 30 min, the aliquots of purified TMEM106B fibrils were applied onto glow-discharged holey carbon-coated grids (C-Flat, 300 mesh, 1.2/1.3, Cat. 71159) and blotted with filter paper and plunge-frozen into liquid ethane, utilizing the Vitrobot Mark IV (FEI, Thermo). Cryo-EM micrographs, consisting of 40 frames per micrograph, were captured using the Thermo Fisher Titan Krios G4 cryo transmission electron microscope, operating at 300 kV, and equipped with a BioContinuum K3 direct detector (Gatan). Energy filter (GIF Quantum) with a 20 V slit width was employed to eliminate inelastically scattered electrons. Super-resolution movies were recorded at a magnification of × 105,000, featuring a pixel size of 0.83 Å pixel-1, and an approximate total dose of about 55 e/Å [2] over an exposure time of 2 s. Automated cryo-EM data collection was executed using EPU software (Thermo), with defocus values ranging from −1.0 to −2.4 μm.

### Helical reconstruction

Movie frames were gain-corrected, aligned, dose-weighted, and summed using MotionCor implemented in RELION [55]. For the brain-extracted fibrils from case 1 of PDD patient, initially, 5875 fibrils from 6822 micrographs were manually picked. These fibrils were segmented into box size in 288 pixels with an inter-box distance of 23.9 Å. Subsequently, re-extraction of these particles using a 960 pixel box size and downscaled them to 360 pixels was applied. Reference-free two-dimension classification was performed with a regularization parameter (*T* = 2), employing a decreasing in-plane angular sampling rate (0.5° and 0.2°), along with a descending offset search range (10 pixels and 5 pixels) and step (1 pixel and 0.5 pixels).

The purified segments underwent further three-dimensional (3D) classification with an initial model (EMD-33054) derived from our previous work [34]. 3D auto-refinements and 3D classifications were combined and employed to select the highest-quality segments. To enhance the resolution of the 3D reconstruction map, Bayesian polishing and contrast transfer function refinements and 3D auto-refinements were conducted. Subsequently, the maps were sharpened using a soft-edge solvent mask through the standard post-processing program in RELION 3.1 [55]. The 0.143 Fourier shell correlation criterion was used to estimate the resolution of 3D density map. Local resolution was assessed using the Local resolution procedure in RELION 3.1, employing the same mask and B-factor as in post-processing.

### Atomic model building

The atomic model of TMEM106B fibril derived from the case 1 of PDD patient was constructed in accordance with the post-processing density map. The atomic model of case 1 of PDD patient-derived TMEM106B fibril was built based on the map and structure model of Type 1 fibril (PDB ID: 7X83) that we previously reported in COOT [56]. Subsequently, a three-layer model was generated in Chimera and refined using the real-space refinement program within PHENIX [57]. Additional comprehensive details can be found in Table S1.

### Statistical analysis

Statistical analyses were performed using GraphPad Prism 9 (GraphPad Software, San Diego, CA). To evaluate the burden of TMEM106B fibril, IHC of brain sections from all cases of this study were performed using anti-TMEM106B (NOVUS, Cat. NBP1-91311), and corresponding images were scanned using Pannoramic Scan (3DHISTECH). Six different standard 40× microscopic fields were randomly selected from sections of each case and were graded by quantifying the percentage of immunoreactive positive cells in each visual field using the “cell counter” tool of ImageJ by an investigator blinded to the identity of each image. Statistical significance was analyzed by unpaired *t* test. The significance level was set at *P* < 0.05 (two-sided).

## Supplementary Material

lnae011_suppl_Supplementary_Material

## Data Availability

Cryo-EM map and corresponding refined atomic model of TMEM106B fibril derived from case 1 of PDD have been deposited in the Electron Microscopy Data Bank (EMDB) under accession number 38069 and the Protein Data Bank (PDB) under accession number 8X5H, respectively. Additional data supporting findings of this study are available within the article and supplementary materials.
