# Supplementary material for: Tracing TMEM106B fibril deposition in aging and Parkinson’s disease with dementia brains
Source: Life Med. 2024 Mar 7;3(1):lnae011. doi: 10.1093/lifemedi/lnae011 (PMC11749594; doi:10.1093/lifemedi/lnae011)
Supplement: lnae011_suppl_Supplementary_Material [file lnae011_suppl_Supplementary_Material.docx]

**Supplementary information**

**Tracing TMEM106B Fibril Deposition in Aging and Parkinson’s Disease with Dementia Brains**

Wanbing Zhao ^1,¶^, Yun Fan^1,¶^, Qinyue Zhao^2^, Zhen Fan^3^, Jue Zhao^1^, Wenbo Yu^1^, Wensheng Li^4^, Dan Li^2,5,6^, Cong Liu^7,8,^*, Jian Wang^1,^*

^1^Department of Neurology and National Research Center for Aging and Medicine & National Center for Neurological Disorders, State Key Laboratory of Medical Neurobiology, Huashan Hospital, Fudan University, Shanghai 200040, China

^2^Bio-X Institutes, Key Laboratory for the Genetics of Developmental and Neuropsychiatric Disorders (Ministry of Education), Shanghai Jiao Tong University, Shanghai 200030, China

^3^Department of Neurosurgery, Huashan Hospital, Shanghai Medical College, Fudan University, Shanghai 200040, China

^4^Department of Anatomy and Histoembryology, School of Basic Medical Sciences, State Key Laboratory of Medical Neurobiology and MOE Frontiers Center for Brain Science, Institutes of Brain Science, Fudan University, Shanghai 200032, China

^5^Zhangjiang Institute for Advanced Study, Shanghai Jiao Tong University, Shanghai 200240, China

^6^WLA Laboratories, World Laureates Association, Shanghai 201203, China

^7^Interdisciplinary Research Center on Biology and Chemistry, Shanghai Institute of Organic Chemistry, Chinese Academy of Sciences, Shanghai 201210, China

^8^State Key Laboratory of Chemical Biology, Shanghai Institute of Organic Chemistry, Chinese Academy of Sciences, Shanghai 200032, China

^¶^These authors contributed equally to this work.

*Correspondence: liulab@sioc.ac.cn (C.L.), wangjian_hs@fudan.edu.cn (J.W.)


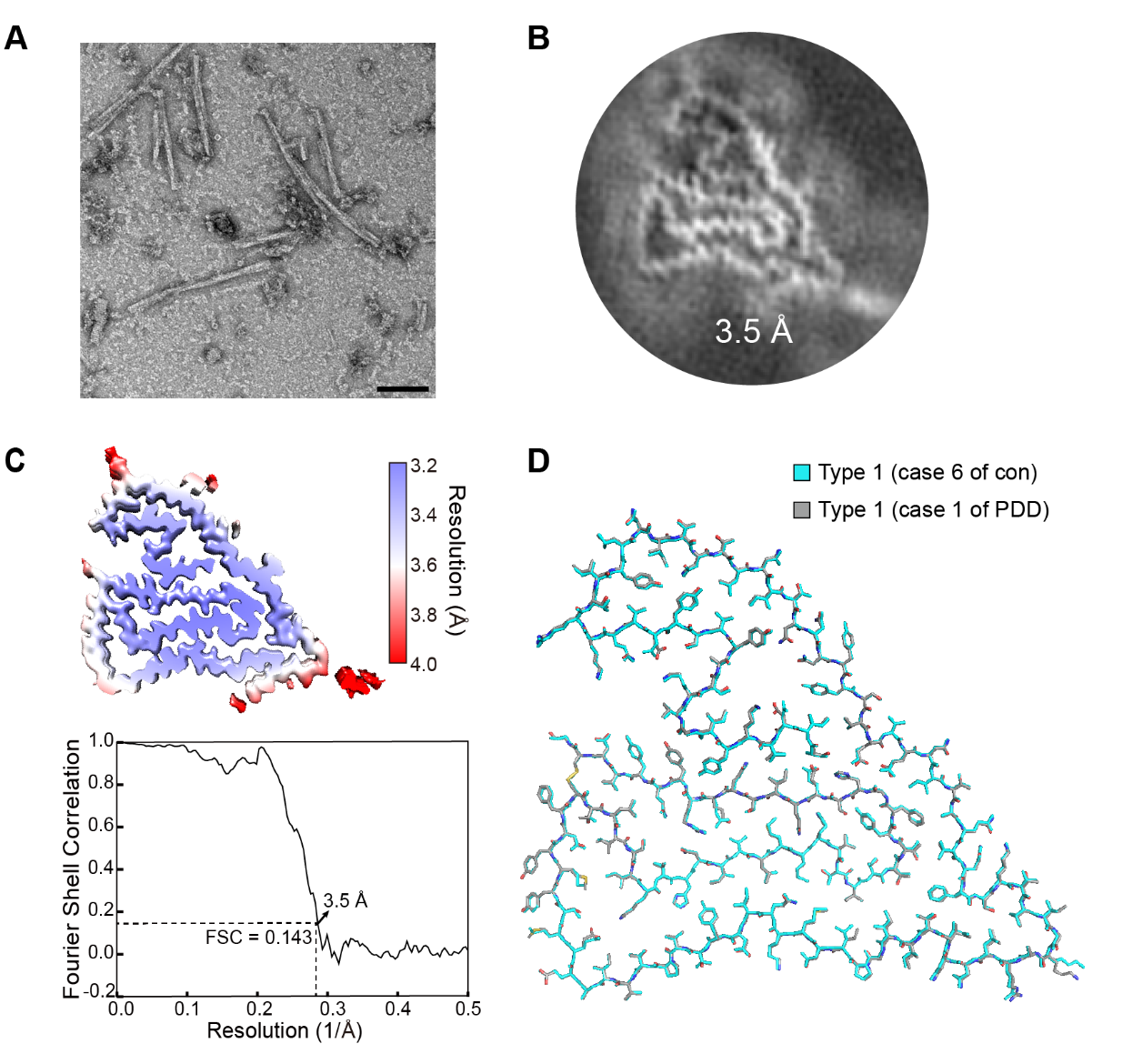


**Figure S1. Cyro-EM structure determination of TMEM106B fibril derived from case 1 of PDD**. The NS-TEM represent image (A) and 3D density map with resolution labeled (B) of TMEM106B fibril extracted from the frontal cortex of case 1 of PDD patient. (C) Local resolution estimation of the reconstruction (top) and gold standard Fourier shell correction curve (bottom) for case 1 of PDD derived TMEM106B fibril. Its overall resolution of is 3.5 Å. (D) Overlay of the atomic models of the Type 1 TMEM106B fibril derived from case 1 of PDD and Type 1 TMEM106B fibril (PDB ID: 7X83) from case 6 of non-ND (corresponding to normal 2 case in our previously study) as we reported^1^ with a root-mean square deviation (RMSD) of 0.280 Å over 135 C-α atoms, which indicates that the *ex vivo* TMEM106B fibril from case 1 of PDD patient corresponds to the Type 1 TMEM106B fibril structure. Scale bar,100 nm (A).

**
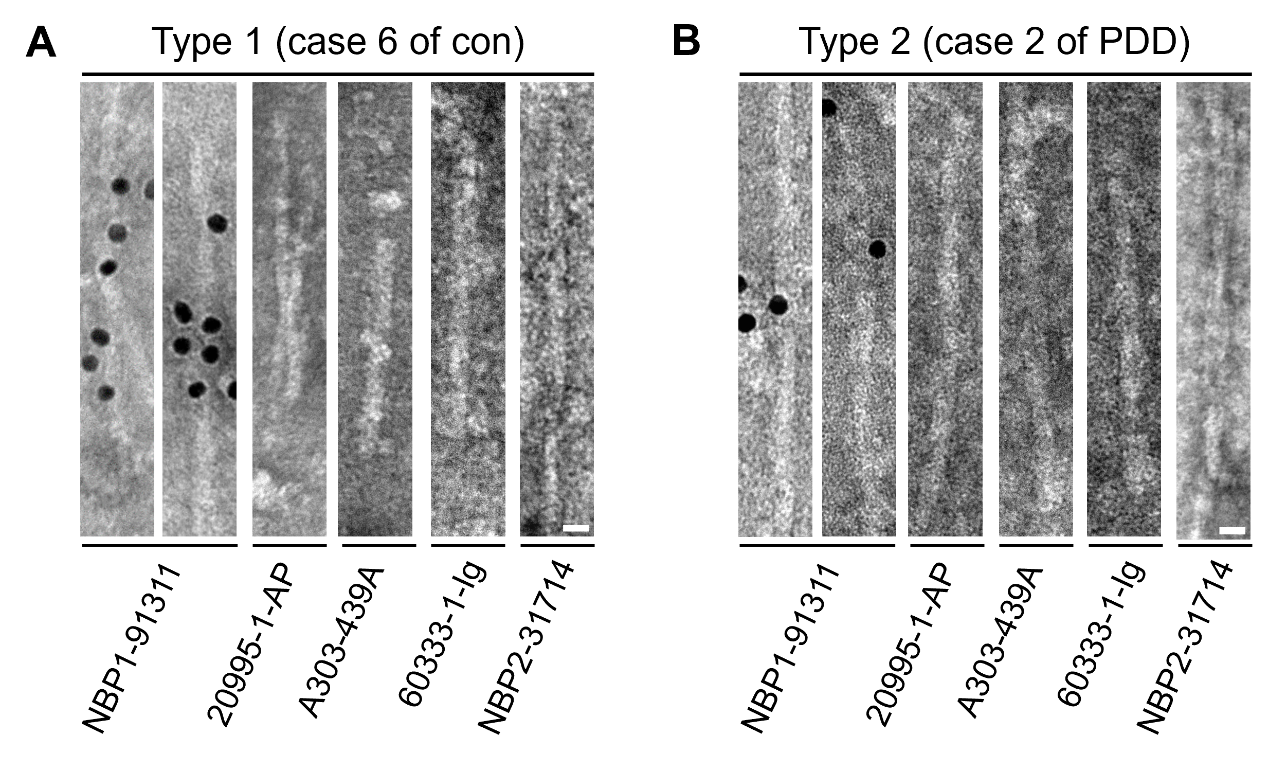
Figure S2. Immunogold staining of TMEM106B fibrils derived from two other cases.** Representative images of immunogold staining of Type 1 TMEM106B fibril extracted from case 6 of non-ND control (A) and Type 2 TMEM106B fibril extracted from case 2 of PDD (B) using five commercial antibodies. Scale bar, 50 nm (A and B).


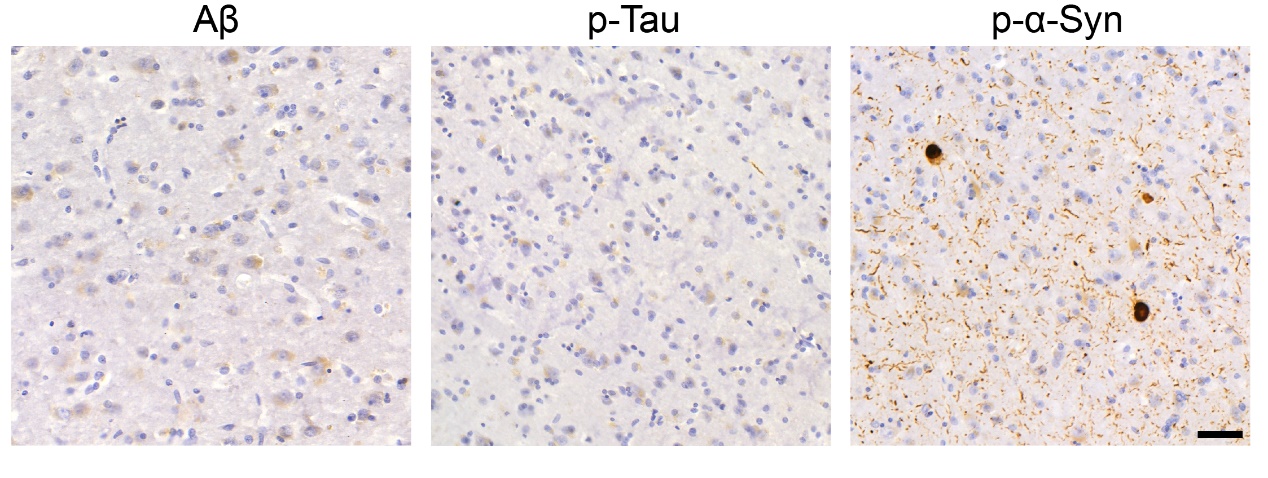


**Figure S3. Pathological characterization of case 1 of PDD.** IHC staining of abundant p-α-syn pathology, including Lewy bodies and Lewy neurites, but no p-Tau pathology and Aβ plaques in the cingulate cortex of case 1 of PDD patient. Scale bar, 50 μm.


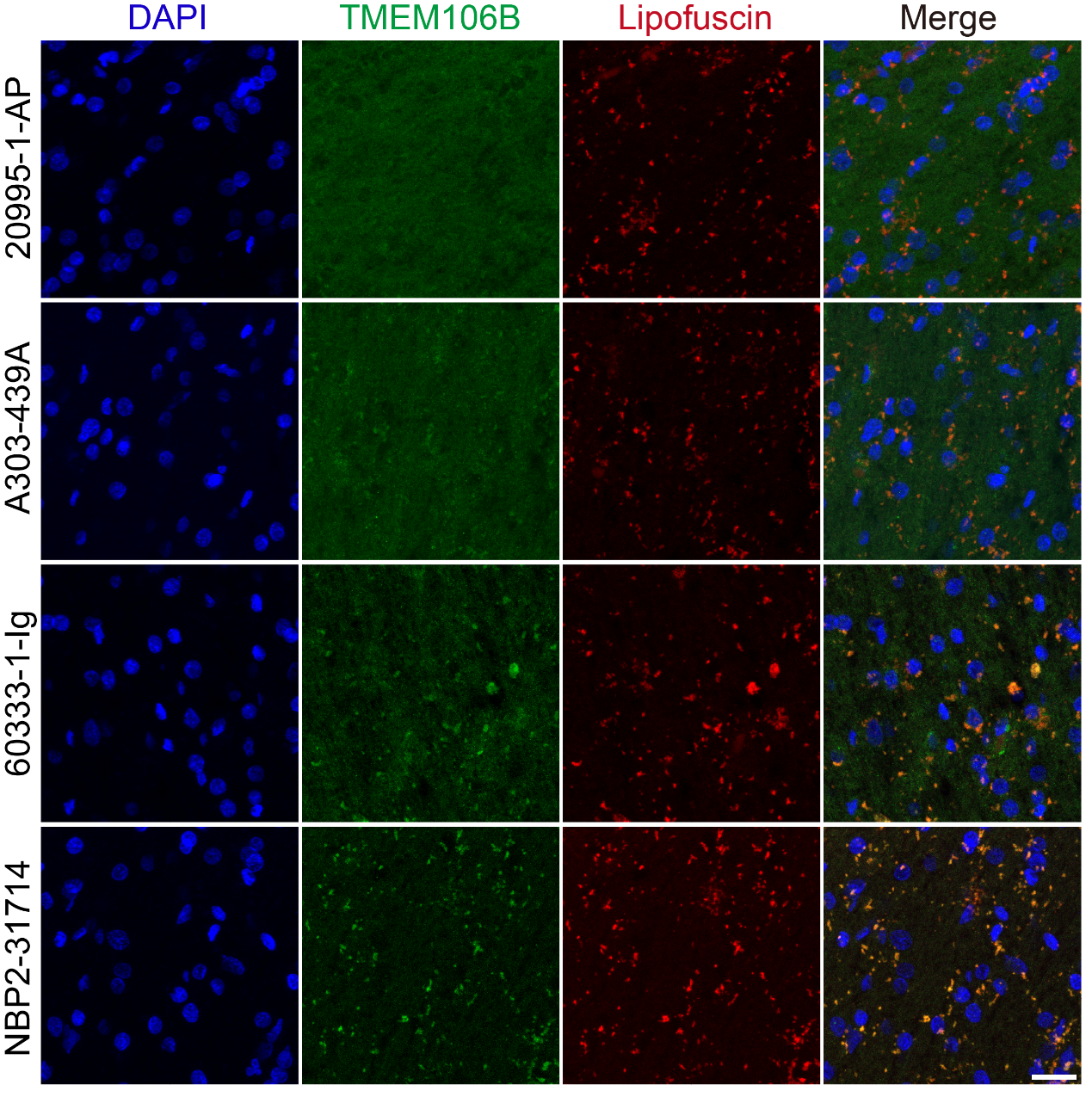


**Figure S4. IF staining of TMEM106B in brain slices with heat pre-treatment in sodium citrate.** The brain slices of the frontal cortex from case 1 of PDD were used. The intensity of autofluorescent lipofuscin was captured in a separate channel (red). Scale bar, 25 μm.

**Table S1. Cryo-EM structural determination and**

**model statistics**

| Data collection and processing | **TMEM106B**  (EMD-38069)  (PDB: 8X5H) |
| --- | --- |
| **Data Collection**  Magnification | 105,000 |
| Pixel size (Å) | 0.83 |
| Defocus Range (μm) | −1.0 to −2.0 |
| Voltage (kV) | 300 |
| Camera | BioContinuum K3 |
| Microscope | Krios G4 |
| Exposure time (s/frame) | 0.05 |
| Number of frames | 40 |
| Total dose (e^−^/Å^2^) | 55 |
| **Reconstruction** |  |
| Micrographs | 6,822 |
| Manually picked fibrils | 5,875 |
| Box size (pixel) | 360 |
| Inter-box distance (Å) | 29.88 |
| Initial particle images (no.) | 52,232 |
| Final particle images (no.) | 2,165 |
| Resolution (Å) | 3.5 |
| Map sharpening B-factor (Å^2^) | −59.03 |
| Helical rise (Å) | 4.83 |
| Helical twist (° ) | −0.70 |
| **Atomic model** |  |
| Non-hydrogen atoms | 3,258 |
| Protein residues | 405 |
| Ligands | 0 |
| RMSD bond lengths | 0.007 |
| RMSD bond angles | 0.75 |
| All-atom clash score | 15.21 |
| Rotamer outliers (%) | 0.00 |
| Ramachandran outliers (%) | 0.00 |
| Ramachandran allowed (%) | 13.53 |
| Ramachandran favored (%) | 86.47 |

EMDB, Electron Microscopy Data Bank; PDB, Protein Data Bank.

**Reference**

1 Fan Y, Zhao Q, Xia W *et al.* Generic amyloid fibrillation of TMEM106B in patient with Parkinson's disease dementia and normal elders. *Cell research* 2022; **32**:585-588.
